# A randomized controlled trial evaluating the short-term efficacy of a single-administration intramuscular injection with the fixed combination of thiocolchicoside-diclofenac versus diclofenac monotherapy in patients with acute moderate-to-severe low back pain

**DOI:** 10.1186/s12891-023-06599-0

**Published:** 2023-06-10

**Authors:** Konstantinos Iliopoulos, Panagiota Koufaki, Stavros Tsilikas, Kyriakos Avramidis, Antonios Tsagkalis, Clio Mavragani, Elias Zintzaras

**Affiliations:** 1 Orthopaedic Department, General Clinic “Eftychios Patsidis”, Larissa, Greece; 2 WinMedica S.A, 1-3 Oidipodos Str., & Attiki Odos Turnoff 33-35, 15238 Chalandri, Athens Greece; 3 Orthopaedic Department, Central Clinic of Athens, Athens, Greece; 4 Orthopaedic Department, General Hospital of Larissa, Larissa, Greece; 5 Orthopaedic Department, IASO Thessaly, Larissa, Greece; 6 Department of Physiology, School of Medicine, National and Kapodistrian University of Athens, Athens, Greece; 7 BECRO, Athens, Greece; 8 Department of Biomathematics, University of Thessaly School of Medicine, Larissa, Greece; 9 Pharmacology & Drug Development Program, Sackler School of Graduate Biomedical, Tufts University School of Medicine, SciencesBoston, MA USA

**Keywords:** Acute, Low back pain, Diclofenac, Thiocolchicoside, NSAID

## Abstract

**Background:**

Non-specific acute low back pain (LBP) is a common health problem that may be accompanied by muscle spasm and decreased mobility. The combination of non-steroidal anti-inflammatory drugs and muscle relaxants represents an advantageous therapeutic option, however, available data on their combined use are conflicting.

This prospective, randomized, single-blind, two-parallel-group trial assessed the efficacy of a single intramuscular (IM) injection of the fixed-dose combination (FDC) diclofenac (75 mg)-thiocolchicoside (4 mg/4 ml) product (test treatment) compared to diclofenac (75 mg/3 ml) alone (reference treatment) for the symptomatic relief of acute LBP. Tolerability and safety were also assessed as secondary variables.

**Methods:**

One hundred thirty-four patients were enrolled (safety population) and randomly allocated to the combination or single-agent regimen. Pain intensity and muscle spasm, assessed respectively by the patient-reported visual analogue scale and investigator-performed finger-to-floor distance test, were determined prior to the injection as well as 1 and 3 h post-injection in 123 patients (per-protocol population). The patients were blinded to treatment. Safety was assessed up to 24 h post-injection.

**Results:**

The test treatment was superior in both alleviating the pain intensity and reducing the finger-to-floor distance at both 1 (*p* < 0.01 and *p* = 0.023 respectively) and 3 h post-injection (*p* < 0.01). A higher percentage of patients experienced > 30% reduction in pain intensity at 1 and 3 h with the test treatment (*p* = 0.037 and *p* < 0.01 respectively). The corresponding VAS (SD) scores for the test treatment group were at baseline, 1 and 3 h post-injection 72.03 (± 11.72), 45.37 (± 16.28) and 31.56 (± 15.08) respectively and for the reference treatment group 65.20 (± 12.16), 48.98 (± 18.76) and 44.52 (± 17.33) respectively. No adverse effects were reported with the combination treatment, whereas two patients treated with diclofenac reported dizziness.

**Conclusions:**

The FDC treatment is an effective and well-tolerated option for the symptomatic treatment of LBP. Clinical and patient-reported assessments confirmed that a single IM injection of FDC diclofenac-thiocolchicoside was more effective than diclofenac alone in conferring rapid and sustained improvement in mobility and pain intensity.

**Trial registration:**

EudraCT No: 2017–004530-29 Available at https://eudract.ema.europa.eu/ Registered 04 Dec 2017.

**Supplementary Information:**

The online version contains supplementary material available at 10.1186/s12891-023-06599-0.

## Background

Low back pain (LBP) is a common health problem throughout the world, with an estimated yearly global prevalence of 38% [1]. It is the leading cause of living with disability for years and has profound psychological and socio-economic impacts on the lives of the affected patients [2–4]. LBP may be attributed to specific causes such as trauma, cancer, or systemic disease; however, in 90% of cases a specific pathoanatomical cause cannot be determined and patients are diagnosed with non-specific LBP [5].

Acute episodes of non-specific LBP can be triggered by physical (e.g., bending, twisting, lifting) and/or psychosocial (e.g., tiredness, stress, anxiety, depression) factors [6]. Besides pain, muscle spasms and reduced motility are commonly observed in patients who present with acute non-specific LBP [7]. Although these symptoms are usually self-limiting, the majority of patients experience a recurring episode of acute LBP within a year [8]. The spasm-pain-spasm cycle is a generally accepted theory, which suggests that muscle spasms caused by an initial event, such as injury, can induce pain and reduced range of motion. The feeling of pain can cause further muscle contractions that further increase the intensity of pain, resulting in the self-perpetuation of the cycle and chronicity of LBP [9, 10].

Alleviating the intensity of pain and improving the mobility and physical function of patients are the main therapeutic goals of acute LBP management [11]. In routine clinical practice, patients with acute LBP are not hospitalized; they are usually treated as outpatients and administered injectable treatments for short-term relief followed by instructions for at-home treatment. Non-steroidal anti-inflammatory drugs (NSAIDs), such as diclofenac, are part of the first-line armamentarium for the short-term treatment of acute LBP, owing to their proven clinical efficacy and acceptable tolerability profile [12]. Skeletal muscle relaxants are also effective for the short-term management of acute LBP and are the third most commonly prescribed adjuvant drug for LBP [11, 13–15]. This broad class of drugs includes non-benzodiazepine antispasmodics and antispastics [15]. Recommendations for the use of muscle relaxants in clinical practice guidelines vary [12, 15]. Various systematic reviews have compared muscle relaxants, as monotherapy or in combination schemes, versus placebo or other modalities, including but not limited to analgesics, NSAIDS, and have provided evidence that muscle relaxants are effective for the short-term symptomatic relief of LBP (acute or chronic) [14–16]. However, they must be used with caution due to the high risk of adverse events, such as drowsiness and dizziness [14–16].

The combined use of NSAIDs and skeletal muscle relaxants, both of which can be administered orally or intramuscularly (IM), can target simultaneously both of the components of the spasm-pain-spasm cycle, and thus, represents an advantageous therapeutic option in the treatment of acute LBP compared to either agent alone [17–20]. Various studies have investigated the effect of the addition of muscle relaxants to NSAIDs compared to NSAID monotherapy for LBP with conflicting results, although the majority of evidence suggests that the combination scheme is more effective than NSAID alone [18, 21–26]. The complementary actions of muscle relaxants and NSAIDs have, thus, been combined in single fixed-dose combination (FDC) oral or IM-administered products to effectively relieve pain and muscle spasms in patients with acute LBP compared to NSAID alone [21, 26]. The timely management and treatment of LBP are essential to reduce the patient’s pain and restore physical functioning. Particularly the IM-administered FDCs are also expected to reduce the discomfort, cost and potential risks of multiple injections of the individual agents [26].

Thiocolchicoside, a well-known competitive GABA_A_ receptor antagonist, is a non-sedating muscle relaxant with anti-inflammatory and analgesic effects that has been used for the symptomatic relief of LBP following oral or IM administration [17, 19, 20, 27, 28]. Although thiocolchicoside monotherapy for acute LBP versus placebo was shown to be effective since the first day of administration, thiocolchicoside 4 mg/2 ml IM injection (Muscoril®) can only be administered as adjuvant treatment for LBP according to the currently approved indications. Diclofenac is a potent NSAID. Following IM injection of 75 mg/3 ml diclofenac (Voltaren®), a pronounced analgesic effect in moderate and severe pain is exerted, which sets in within 15 to 30 min, making diclofenac IM injection particularly suitable for the initial treatment of acute LBP [29–31]. According to the approved labelling information, diclofenac IM injection can be administered at a maximal dose of 75 mg twice daily no more than 2 days, and if necessary, treatment can continue with diclofenac tablets or suppositories [31].

Clinical evidence has confirmed that the combination of thiocolchicoside and NSAIDs was more effective than NSAID alone in the symptomatic treatment of acute LBP for both pain relief and muscle spasm [17, 19, 20, 27]. Based on the posologies of the individual agents that are used in routine clinical practice, FDC diclofenac (75 mg, as diclofenac sodium)-thiocolchicoside (4 mg/4 mL) solution for IM injection (FDC diclofenac-thiocolchicoside henceforward) has been developed by Win Medica S.A. for the symptomatic management of acute LBP and confers the advantage of limiting the number of injections of the individual agents to afflicted patients. Based on the evidence by Sproviero et al., the FDC of diclofenac-thiocolchicoside was recently shown to be as effective as the separate injection of the individual agents in the treatment of patients with non-specific moderate-to-severe acute LBP following once-daily IM administration for 5 days, while improving patient compliance and tolerability, due to the decreased number of injections [26]. However, there is a paucity of publications on the effect of IM administered FDC diclofenac-thiocolchicoside on acute LBP shortly after administration. Besides, in routine clinical practice, patients with acute LBP are not hospitalized; they are usually treated as outpatients and administered injectable treatments for short-term relief followed by instructions for at-home treatment. In the present randomized, single-blind, two-parallel-group trial, we sought to evaluate and compare the efficacy and tolerability of FDC diclofenac-thiocolchicoside (test treatment) versus diclofenac 75 mg/3 ml (as diclofenac sodium, reference treatment) monotherapy, following a single intragluteal administration, in the symptomatic relief of patients with non-specific moderate-to-severe acute LBP.

## Methods

### Trial design and participants

This prospective, randomized, single-blind, two-parallel-group trial was conducted in nine hospitals and clinics in central Greece following approval by the National Ethics Committee and the National Organization for Medicines (EudraCT No: 2017–004530-29). All participants provided informed consent at recruitment, which was performed in accordance with the ethical standards of the Declaration of Helsinki. The participants were blinded to the treatment they received.

Adult patients were enrolled in the current trial if they presented with acute, moderate to severe LBP, defined as ≥ 40 mm on the visual analogue scale (VAS) range of 0–100 mm, ≤ 7 days prior to screening. Patients with the following characteristics were excluded from the trial: 1) back pain due to metastatic cancer spinal infection or spinal cord compression, 2) treatment with a NSAID or skeletal muscle relaxant within the last 24 h prior to trial entry, 3) history of inflammatory bowel disease, 4) history of peptic ulceration, gastrointestinal bleeding, or severe dyspepsia, 5) history of concurrent systemic disease, 6) history of thrombopenia, easy bruising, hemophilia, or deficiency of coagulation factors, 7) history of hepatic or renal failure, 8) history of established congestive heart failure (NYHA II-IV), ischemic heart disease, peripheral arterial or cerebrovascular disease, 9) known allergy to NSAIDs and skeletal muscle relaxants, 10) poorly controlled arterial hypertension, 11) asthma or other allergic disorders induced by acetylsalicylic acid or NSAIDs, 12) concurrent administration of angiotensin converting enzyme inhibitors for arterial hypertension, antiplatelet agents, or anticoagulants, 13) pregnant, breast-feeding, under a highly effective contraceptive method of birth control, 14) current participation or prior participation within the last 30 days prior to trial entry in another investigational device or drug trial, patients, 15) inability or unwillingness to comply with the trial procedures, and 16) legally incapacitated or legally institute-detained patients.

Sample size calculation was performed with the StudySize software (version 2.0.5, CreoStat HB, 2012). The required sample size to observe a difference of 10% in mean change in VAS from baseline to 3 h after administration between Test and Reference products which is considered as clinically significant [28, 32], assuming a common standard deviation of 17 [28], with a statistical power 90% and two-sided significance level 5% was at least 122 patients (equally distributed/randomized to the two treatment groups). Based on an estimated drop-out rate of 10%, a sample size of 134 participants was required to detect a difference of 10% in the mean change of VAS from baseline at 3 h post-administration.

### Procedures-evaluations

Pre-intervention evaluation included patient self-reported pain intensity assessment at rest using the VAS score (scoring range 0–100 mm), with higher scores indicating greater pain (“0 = No pain”; “100 = Worst imaginable pain”) and investigator evaluation of the muscle spasm using the finger-to-floor distance test. VAS scores were assigned individually by each patient. Other baseline procedures included the collection of patient sociodemographic and medical history data.

The patients were then randomized 1:1 using permuted block randomization (FORTRAN90 IMSL) per site to receive a single deep intragluteal injection into the upper outer quadrant of either the test treatment (FDC diclofenac-thiocolchicoside) or the marketed solution containing 75 mg/3 ml of diclofenac (reference treatment, Voltaren®/Novartis). The assignment of treatment to patients according to the randomization scheme was operated centrally by the CRO that performed clinical monitoring. A separate randomization scheme was adopted for each site (i.e. randomization stratified by site). The randomization was performed using permuted blocks, i.e. random sequences of treatment allocations that contain the two treatments (A = investigational medicinal product or B = comparator) in a 1:1 ratio. Within a block, the sequence of treatment allocations was chosen at random from all possible permutations. The treatment allocation procedure consisted of preparing lists of permuted blocks for each center and reading off the next treatment allocation (Α-Test or Β-reference) from that list. Patients were allocated to treatments according to the screening number in ascending order. The personnel that made all contacts with patients and performed all clinical trial-related examinations was blinded to the block length with no access to the randomization scheme. The randomization scheme per site is included in the Supplementary Material.

Then, pain intensity assessment using VAS by the patient and muscle spasm assessment by the investigator were performed at Intermediate (1 h post-injection) and Test of Cure visit (3 h post-injection). The investigators who administered the treatment also performed the clinical assessments. The efficacy assessments that were performed in this clinical trial were those which are traditionally used in patients with LBP. Safety parameters were evaluated at the End of Study Visit (24 h post-injection).

The primary endpoint was the mean change in VAS from baseline to 3 h in subjects treated with the test treatment as compared to subjects treated with the reference treatment. However, in the analysis, the change from 3 h to baseline was used. Secondary outcome measures included the 1) mean change from baseline in the VAS score at 1 h post-administration, 2) the percentage of patients demonstrating > 30% reduction in pain intensity (as assessed via VAS score) at 1 and 3 h post-administration compared to baseline, 3) muscle spasm improvement assessed via the mean change of finger-to-floor distance from baseline at 1 and 3 h post-administration, and 4) the percentage of patients experiencing adverse events and withdrawn from the trial for safety reasons related to treatment. For the purpose of this manuscript, muscle spasm assessments (endpoint 3) are synoptically referred to, in order to minimize the potential introduction of bias from the effect of the test treatment (due to the presence of the muscle relaxant thiocolchicoside) versus the reference treatment.

Based on literature review, an expert panel and a workshop during the “VIII International Forum on Primary Care Research on Low Back Pain” (Amsterdam, June 2006), for a range of commonly used back pain outcome measures, including VAS scale 0–100, a 30% improvement from baseline has been proposed as a useful threshold for identifying clinically meaningful improvement [32]. The patients remained at the sites until the Test-of-cure visit.

### Statistical analyses

Efficacy analyses were performed on the per-protocol population which comprised the intent-to-treat patients (ITT) (i.e., all randomized patients with at least one VAS measurement post-baseline) who had no major protocol deviations, completed the VAS measurements within the allowed time frames, and did not take any prohibited concurrent medication (corticosteroids, immunosuppressive, anti-inflammatory, neuromuscular blocking, and pain relief drugs).

The primary efficacy analysis was conducted as a test of superiority using covariance model analysis by estimating the two-sided 95% confidence interval (CI) for the difference (test treatment) in the VAS score changes, with the baseline score being used as the covariate and the treatment score as the factor. The difference between the test and the reference treatment was considered significant at *p* < 0.05. The secondary efficacy variables were analyzed by using either the chi-square test, a log-linear model, or the Fisher’s exact test, depending on the nature of the data. Odds ratios were calculated using logistic regression unadjusted and adjusted for possible effect modifiers, namely age and gender. The parametric independent t-test and/or general linear model, unadjusted and adjusted for possible effect modifiers, were used for the analysis of secondary continuous variables. For the analyses of the demographic and other baseline data, depending on the nature of the data, the parametric independent t-test or the non-parametric Mann–Whitney U-test was used for continuous variables and the chi-square test or the Fisher’s exact test was used for categorical variables. The safety population comprised all patients, who received at least one injection of the test or the reference treatment. Adverse events were summarized according to MedDRA Version 21.1. Safety data were analyzed by using summary statistics (percentages and mean values ± standard deviation). The SPSS software (version 22.0, IBM Corp., 2013) was used for the statistical analyses.

## Results

### Patient disposition

A total of 134 patients with acute LBP were screened and enrolled from 9 hospitals, public and private, of central Greece between 6 March 2018 and 21 May 2019 (due to the well-established nature of the individual active ingredients, all 134 patients who were eligible enrolled the study).

**Fig. 1:**
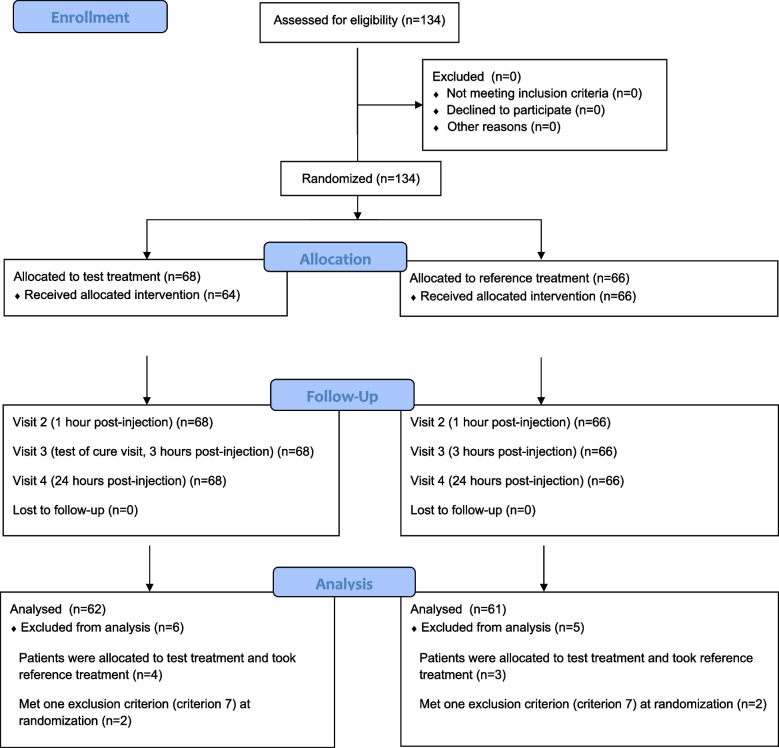
Patient disposition

The patients were randomized to receive the test treatment (*N* = 68) or reference treatment (*N* = 66). All of the participants received the allocated treatment, however, 6 participants in the test group and 5 participants in the reference group were not included in the per-protocol analysis due to protocol violations. Thus, the per-protocol analysis population included *N* = 62 patients for the test treatment and *N* = 61 patients for the reference treatment. Patient disposition is outlined in Fig. 1.

### Baseline patient characteristics

Demographic and clinical features of the enrolled population at baseline are shown in Tables 1 and 2 (per protocol and ITT population respectively). Age was comparable for both treatment groups. Regardless of per protocol or ITT population, slightly over 50% of the patients who were administered the test treatment and a third of the patients who were administered the reference treatment were of male gender (non-statistically significant difference). There were no statistically significant differences in the finger-to-floor distance assessments and the percentage of patients with muscle spasm between treatment groups at baseline, whereas the VAS score was statistically significantly higher in the test group (*p* = 0.002).

**Table 1:** Baseline patient characteristics per treatment group (per protocol population)

Baseline characteristics	Test treatment (*N* = *62*)	Reference treatment (*N* = *61*)	*p*-value
Age, mean (± SD), *years*	52.00 (± 14.88)	51.23 (± 14.51)	0.772
Male, *n%*	33 (53.2%)	23 (37.7%)	0.085
Muscle spasm, *n%*	55 (88.7%)	56 (91.8%)	0.567
Finger-to-floor distance (± SD), *cm*	44.52 (± 17.33)	41.02 (± 16.12)	0.249
VAS score (± SD)	72.03 (± 11.72)	65.20 (± 12.16)	0.002^*^

Definitions: Test treatment: Fixed dose combination of diclofenac 75 mg (as diclofenac sodium)- thiocolchicoside 4 mg/4 ml intramuscular injection; Test treatment: Diclofenac 75 mg/3 ml (as diclofenac sodium) intramuscular injection

*Abbreviations*: *SD* standard deviation, *VAS* visual analogue score

^*^indicates significance compared to reference treatment

**Table 2:** Baseline patient characteristics per treatment group (ITT population)

Baseline characteristics	Test treatment (*N* = *62*)	Reference treatment (*N* = *61*)	*p*-value
Age, mean (± SD), *years*	51.54 (14.91)	51.21 (14.20)	0.895
Male, *n%*	34 (50%)	25 (37.9%)	0.160
Muscle spasm, *n%*	61 (89.7%)	61 (92.4%)	0.320
Finger-to-floor distance (± SD), *cm*	44.21 (16.74)	40.53 (15.73)	0.193
VAS score (± SD)	71.93 (11.31)	65.17 (12.14)	0.001^*^

Definitions: Test treatment: Fixed dose combination of diclofenac 75 mg (as diclofenac sodium)- thiocolchicoside 4 mg/4 ml intramuscular injection; Test treatment: Diclofenac 75 mg/3 ml (as diclofenac sodium) intramuscular injection

*Abbreviations*: *ITT* intent-to-treat, *SD* standard deviation, *VAS* visual analogue score

^*^indicates significance compared to reference treatment

### Efficacy outcomes

Covariance model analysis demonstrated that the FDC diclofenac- thiocolchicoside was superior compared to diclofenac alone in the reduction of pain intensity as evaluated by the VAS score both at the intermediate and final visit (1 and 3 h, respectively, post-injection). The test treatment was also superior compared to the reference treatment in reducing the finger-to-floor distance at both 1 and 3 h post-administration (Table 3).

**Table 3:** Efficacy assessments

**Efficacy outcome**	**Timepoint**	**Test treatment** **(*N* = *62*)**	**Reference treatment (*N* = *61*)**	**Treatment Difference** (95% CI)^*^	***p*-value**
VAS score, mean (± SD)	Baseline	72.03 (± 11.72)	65.20 (± 12.16)	-	
	1 h	45.37 (± 16.28)	48.98 (± 18.76)	-10.032 (-15.073 to -4.990)	< 0.01^†^
	3 h	31.56 (± 15.08)	44.52 (± 17.33)	-14.654 (-20.450 to -8.857)	< 0.01^†^
Finger-to-floor-distance, mean (± SD)	Baseline	44.52 (± 17.33)	41.02 (± 16.12)		
	1 h	32.21 (± 16.64)	33.13 (± 16.26)	-3.794 (-7.055 to -0.534)	0.023^†^
	3 h	25.55 (± 13.85)	30.10 (± 14.76)	-6.907 (-10.081 to -3.733)	< 0.01^†^

Definitions: Test treatment: Fixed dose combination of diclofenac 75 mg (as diclofenac sodium)-thiocolchicoside 4 mg/4 ml intramuscular injection; Test treatment: Diclofenac 75 mg/3 ml (as diclofenac sodium) intramuscular injection

*Abbreviations*: *CI* confidence interval, *SD* standard deviation, *VAS* visual analogue scale

^*^indicates significance compared to reference treatment

^†^treatment difference scores were estimated from the difference in the mean changes compared to baseline, as estimated by covariance model analysis, between the test and the reference group. The difference was considered significant if *p* < 0.05

Treatment with the FDC diclofenac-thiocolchicoside resulted in a statistically significantly greater number of patients experiencing > 30% reduction in pain intensity as compared to the treatment with diclofenac alone (Table 4). The odds (both unadjusted and adjusted odds for age and gender) for attaining > 30% reduction in pain intensity compared to baseline were 1.9-fold higher at 1 h and eightfold higher at 3 h post-injection for the test treatment.

**Table 4:** Percentage of patients attaining > 30% reduction in pain intensity

**Efficacy outcome**	**Timepoint**	**Test treatment** **(*Ν* = 62)**	**Reference treatment (*Ν* = 61)**	***p*-value**	**Unadjusted OR** **(95% CI)**	**Adjusted OR** **(95% CI)**^†^
> 30% reduction in pain intensity, *(n%)*	1 h	35 (56.5%)	23 (37.7%)	0.037^*^	1.949 (0.980 to 3.876)	1.914 (0.937 to 3.910)
	3 h	57 (91.9%)	35 (57.4%)	< 0.01^*^	8.469 (2.977 to 24.093)	8.100 (2.803 to 23.403)

Definitions: Test treatment: Fixed dose combination of diclofenac 75 mg (as diclofenac sodium)- thiocolchicoside 4 mg/4 ml intramuscular injection; Test treatment: Diclofenac 75 mg/3 ml (as diclofenac sodium) intramuscular injection

*Abbreviation*: *CI* confidence interval, *OR* odds ratio

^*^indicates significance compared to reference treatment

^†^adjusted for age and sex

### Safety outcomes

The safety population patients included all patients who were exposed to the test or reference product once (*N* = 134). No adverse events were reported in the patients who were administered the test treatment. There were two patients (1.5% of trial patients, 3.03% of reference product) who were presented with dizziness, which was mild in severity, in the diclofenac arm. This adverse event occurred soon after administration in both patients, was considered possibly related to treatment, and spontaneously resolved quickly thereafter. There were no withdrawals from the scheduled assessment due to adverse events.

## Discussion

This phase III, prospective, single-blind, parallel-group trial evaluated the efficacy of the single IM administration of FDC diclofenac-thiocolchicoside compared to diclofenac IM injection alone for the symptomatic relief of Greek patients in non-specific moderate to severe acute LBP. The trial population was representative of the typical patients with non-specific moderate-to-severe LBP, with the mean age and sex ratio of participants being similar to those observed in other clinical and real-life studies [26, 33]. There was no statistically significant difference in the baseline characteristics of the two treatment groups in terms of age, gender, prevalence of muscle spasms, and finger-to-floor distance. Patient-assessed pain, based on VAS score, was greater at baseline in the test treatment group.

Population-based studies have reported that the overall prevalence of LBP is higher in women compared to men across all age groups with a prevalence ratio of female:male ranging from 1.185 to 1.360 across all age groups [34]. This could account for the higher, though non-statistically significant, prevalence of women in the diclofenac arm. Post-menopausal women are at greater risk for LBP due to accelerated lumbar disc degeneration and spine degeneration [34]. Based on age range, the enrolled population included both menopausal and reproductive-age females (median age 50 years (min: 24, max: 86). The enrollment of females of various age groups represents one of the strengths of the current trial.

The effect of a single IM administration of the test and reference treatment resulted in acute LBP relief, based on VAS assessments, both at 1 h and 3 h post-administration. This was anticipated due to the potency of diclofenac IM administration [29–31]. These findings are, also, in line with the rapid recovery that has been reported in other studies of IM administration of thiocolchicoside [28, 35]. The primary efficacy analysis clearly demonstrated the superiority of the FDC test treatment compared to diclofenac monotherapy at rapidly alleviating the pain of patients with acute LBP, as evaluated by the estimated difference between the mean VAS scores of the two treatment groups at 3 h following treatment administration. Similarly, the secondary efficacy analyses, based both on investigator and patient self-reported assessments, complemented the primary endpoint and contributed further evidence in favour of the consistent efficacy of the combination compared to the reference treatment. Thus, the IM administration of the test treatment was associated with a greater and sustained improvement in mobility as assessed by the finger-to-floor distance test and pain intensity at both 1 h and 3 h post-injection. Also, a higher percentage of patients in the test treatment group experienced a > 30% reduction in pain intensity as soon as 1 h post-injection and at 3 h post-injection. In fact, slightly over 90% of the patients that were administered the test treatment had > 30% reduction in pain intensity at 3 h post-injection.

The results of our trial support the findings of a recent phase 3, randomized, controlled trial which evaluated the efficacy of IM diclofenac and thiocolchicoside, administered either as FDC or free combination, in the treatment of patients with acute non-specific moderate-to-severe LBP. Following once-daily treatment for 5 days, both the FDC and the free combination of diclofenac and thiocolchicoside resulted in similar marked improvements in the VAS score and muscle spasms. Especially on the first day of treatment, the mean VAS score (~ 73 mm) improved approximately by 10 mm [26]. The present trial complements the existing results and contributes further evidence on the effects of a single administration of the test treatment shortly after administration versus the reference treatment alone.

Both treatments were overall well-tolerated in the 24 h post-injection follow-up. No adverse drug reactions were observed in patients receiving the combination treatment, whereas dizziness was reported in two patients who were administered diclofenac. Dizziness is commonly reported following the IM administration of diclofenac [31]. Our data concur with available safety data following the short-term (i.e., for 5 days) IM treatment of patients with acute LBP with either FDC diclofenac-thiocolchicoside or their free combination [26, 28, 30, 36]. Similarly to the current trial, dizziness, administration site conditions, or injection site pain as well as serious adverse drug reactions have not been reported with FDC thiocolchicoside-diclofenac or thiocolchicoside alone in the aforementioned studies.

According to the approved labelling for diclofenac IM injection, it is not to administered more than 2 days and if necessary, treatment can be continued with diclofenac tablets or suppositories. Although the guidelines for acute LBP management per country, nevertheless, it is reported that pharmacological treatments for which efficacy has been demonstrated, such as NSAIDs, only have small to moderate effects at best at the immediate term and short term [12]. Based on the available evidence, NSAIDs and skeletal muscle relaxants may be the best possible drug choices if pharmacotherapy is deemed absolutely necessary for the management of non-specific acute LBP. However, due to the association of pharmacological treatments with the risks of AEs, and since acute LBP will eventually resolve, even without pharmacotherapy, the use of non-pharmacological treatments is highly encouraged in acute LBP treatment guidelines. Therefore, the immediate pain relief and muscle spasm improvement that was observed with no AEs following a single IM injection of the FDC test treatment versus the reference treatment alone represents an effective safe therapeutic strategy that is in line with the proposed guideline suggestions for acute LBP management [12].

Τhe potential of reporting bias in patient-reported pain intensity cannot be ruled out. Τhe non-balanced baseline pain intensity VAS score between the two treatment groups may have influenced subsequent assessments [37]. However, one could argue that in the present trial this imbalance strengthens the case for the superiority of the combination treatment, as it resulted in a greater reduction of pain severity even though the group receiving the combination treatment experienced statistically significantly greater pain severity at baseline. As acute LBP can be a self-limiting condition, any potential placebo-derived effect contributing to the decrease of pain severity or anxiety due to pain could not be ruled out, since a placebo group was not included. As this was a single-blinded trial, the patients were blinded to the treatment they received. The participating investigators who administered the treatment also performed the clinical assessments. Thus, the potential for bias in investigator assessments cannot be ruled out. Nevertheless, the primary endpoint of this trial was the change in patient-reported pain intensity, which was scored by each patient individually. Investigator assessments, along with additional patient self-reported assessments, were secondary endpoints that were used to complement primary efficacy assessments. Additionally, VAS patient-reported assessments are among the most frequently used instruments to determine pain intensity in LBP [14–16, 28, 32, 38]. The advantages of using VAS assessments include its validity, correlation with observed pain behaviour and other self-reported measures of pain intensity and its sensitivity to treatment effects [14–16, 28, 32, 38]. Muscle spasm assessment analysis was also favourable for the test treatment. At Test of Cure visit (3 h post-injection), 16.1% of patients (9/56) in Reference and 38.2% of patients with muscle spasm at baseline (21/55) in Test, presented with improvement (*P* = 0.01).

## Conclusion

The findings of this trial have demonstrated that the single IM injection of FDC diclofenac -thiocolchicoside is superior compared to monotherapy with IM diclofenac in the symptomatic treatment of patients with acute non-specific moderate-to-severe LBP, using both patient- and investigator-based assessments The fixed combination resulted rapidly within 3 h post-administration in statistically significantly greater and sustained improvement in pain intensity and mobility and was well-tolerated. Following the promising preliminary short-term beneficial effects, further double-blinded, double-dummy randomized trials that will investigate both the short- and long-term administration effects of the reference versus the test treatment on a larger cohort of patients will be conducted to verify the current results.

## Supplementary Information

**Additional file 1. **

**Additional file 2. **

## Data Availability

Some of the data supporting the findings and figures in this study are already provided. Data are not publicly available due to ethical restrictions but can be obtained from the authors upon reasonable request.

## Abbreviations

FDC: Fixed-dose combination
LBP: Low back pain
IM: Intramuscular
ITT: Intent-to-treat
NSAID: Non-steroidal anti-inflammatory drug
VAS: Visual analogue scale
