# Supplementary material for: A randomized controlled trial evaluating the short-term efficacy of a single-administration intramuscular injection with the fixed combination of thiocolchicoside-diclofenac versus diclofenac monotherapy in patients with acute moderate-to-severe low back pain
Source: BMC Musculoskelet Disord. 2023 Jun 10;24:476. doi: 10.1186/s12891-023-06599-0 (PMC10257820; doi:10.1186/s12891-023-06599-0)
Supplement: Supplementary file 1 — Additional file 1. [file 12891_2023_6599_MOESM1_ESM.docx]

"The randomization was performed using permuted blocks, i.e. random sequences of treatment allocations that contain the two treatments (A = investigational medicinal product or B = comparator) in 1:1 ratio. Within a block, the sequence of treatment allocations was chosen at random from all possible permutations. The treatment allocation procedure consisted of preparing lists of permuted blocks for each center and reading off the next treatment allocation (Α-Test or Β-reference) from that list. Patients were allocated to treatments according to the screening number in ascending order. The personnel that made all contacts with patients and performed all clinical trial-related examinations was blinded to the block length with no access to the randomization scheme.

The randomization scheme was implemented using a FORTRAN90 IMSL subroutine for random permutations. The randomization scheme is provided in "Supplementary Material".

The randomization scheme per site was:

*Site: 100 ABBABAABBABABAABBABAAABBBAABBBAABAABABBABAABBAABBABAABBABABABAABABBABBAABBAABABA*

*Site: 200 BAABABABBABAABBABABABBAAABABBAABBBAABBAA*

*Site: 300 ABBABAABAABBBBAAAABBBABAABABAABBBABABABA*

*Site: 400 BAABBAABBBAAABBABABABABAABBAAABBBAABBBAA*

*Site: 500 BAABAABBABBAABBABABABBAAABABBBAAABBABAAB*

*Site: 600 BBAABBAABABAABBABBAAAABBBBAAABABABABAABB*

*Site: 700 BAABBAABABBAAABBABABBAABAABBAABBABABBABA*

*Site: 800 AABBBAABBAABBABABABABAABABBABAABBBAABABA*

*Site 900: ABABBAABAABBABBABBAAABBAABBABABAABABBAAB*

*Site 110: ABABBBAABABAABABBAABBAABBABABAABABBAAABB*
